# Conversion Surgery for Advanced Gastric Cancer According to First-Line Treatment Strategy: A Single-Center Experience with a Focused Review of the Literature

**DOI:** 10.3390/cancers18152483

**Published:** 2026-08-02

**Authors:** Jun Kinoshita, Kenta Doden, Kengo Hayashi, Ryota Matsui, Hiroto Saito, Megumi Watanabe, Toshikatsu Tsuji, Daisuke Yamamoto, Noriyuki Inaki

**Affiliations:** Department of Gastrointestinal Surgery, Graduate School of Medical Science, Kanazawa University, 13-1 Takara-Machi, Kanazawa 920-8641, Ishikawa, Japan; k.doden@med.kanazawa-u.ac.jp (K.D.); k-hayashi@staff.kanazawa-u.ac.jp (K.H.); ryota-matsui0818@staff.kanazawa-u.ac.jp (R.M.); masaeritomo16@staff.kanazawa-u.ac.jp (H.S.); mwatanabe@staff.kanazawa-u.ac.jp (M.W.); tsuji104@staff.kanazawa-u.ac.jp (T.T.); yamamoto-daisuke@staff.kanazawa-u.ac.jp (D.Y.); n.inaki@med.kanazawa-u.ac.jp (N.I.)

**Keywords:** gastric cancer, conversion surgery, immune checkpoint inhibitor, trastuzumab, pathological response, oligometastasis, prognostic factors, precision medicine

## Abstract

**Background/Objectives:** First-line therapy for advanced gastric cancer (AGC) has evolved from cytotoxic chemotherapy to HER2-targeted and immune checkpoint inhibitor (ICI)-based regimens, yet conversion surgery (CS) outcomes across these strategies remain poorly characterized. We describe CS outcomes and prognostic factors by first-line strategy at a single center. **Methods:** We retrospectively reviewed 187 patients with AGC who began first-line therapy from 2011, grouped as cytotoxic (CTX, *n* = 127), HER2-targeted (trastuzumab, *n* = 21), or ICI (*n* = 39). CS was defined as resection after response, including an extended oligometastatic definition (*n* = 74). Overall survival (OS) was measured from chemotherapy initiation, and prognostic factors were assessed by Cox regression. **Results:** Median OS was 14.8, 18.9, and 20.9 months for CTX, trastuzumab, and ICI, respectively (*p* = 0.048). CS rates were comparable (41%, 33%, and 38%; *p* = 0.794). Pathological response was more pronounced after trastuzumab/ICI (grade 3 and ypStage 0/1; both *p* < 0.001). Among CS cases, R0 resection (hazard ratio [HR] 0.31) and trastuzumab/ICI therapy (HR 0.37) were independent favorable factors, whereas high inflammatory–nutritional indices (NLR, CAR) were independent poor prognostic factors. OS was comparable between oligometastatic and conventional CS (*p* = 0.324). **Conclusions:** In this hypothesis-generating study, response depth tracked tumor biology, whereas survival was determined by R0 resection, targeted/ICI therapy, and host inflammatory–nutritional status—two largely dissociable axes informing biology- and host-based selection of CS candidates for prospective testing.

## Simple Summary

For patients with advanced gastric cancer that cannot be removed by surgery at diagnosis, drug therapy can sometimes shrink the disease enough to make an operation called conversion surgery possible. First-line treatments have expanded from conventional chemotherapy to HER2-targeted drugs and immunotherapy, but the outcomes of conversion surgery across these different strategies are not well described. We reviewed 187 patients treated at a single center, grouped according to their first-line treatment, and examined how often they underwent conversion surgery, their surgical and pathological outcomes, and the factors linked to survival. We also summarized previously published reports to place our findings in context. Complete tumor removal and the type of drug therapy were associated with longer survival, while the patients’ general nutritional and inflammatory conditions reflected their overall outlook. These descriptive, single-center findings may help identify patients who are most likely to benefit from conversion surgery.

## 1. Introduction

Gastric cancer remains a leading cause of cancer incidence and mortality worldwide [1], and systemic pharmacotherapy is the mainstay of treatment for patients with unresectable advanced or metastatic disease [2,3]. Although the prognosis of advanced gastric cancer (AGC) remains poor, recent advances in systemic therapy have improved treatment responses; some patients with initially unresectable stage IV disease now achieve substantial tumor reduction.

First-line therapy for AGC has shifted from fluoropyrimidine-plus-platinum cytotoxic chemotherapy to biomarker-driven precision medicine. Trastuzumab-based therapy has become the standard of care for HER2-positive disease [4]. Subsequently, the addition of immune checkpoint inhibitors (ICIs)—nivolumab or pembrolizumab—to chemotherapy improved overall survival (OS), particularly in patients with a high PD-L1 combined positive score (CPS), and has become a first-line standard [5,6,7]. In addition, the upfront combination of ICIs with anti-HER2 therapy for HER2-positive disease [8] and zolbetuximab for Claudin18.2-positive disease [9,10] have diversified the therapeutic targets. Real-world data from Japan also support the efficacy and safety of first-line ICI combinations [11]. In pivotal first-line trials, these biomarker-driven regimens have achieved response rates of approximately 50–60%, with a median duration of response exceeding 8–10 months [5,6,7], and complete responses are now occasionally observed. As a result, a window has emerged in which surgical intervention with curative intent can be considered for patients who achieve durable systemic disease control [12].

Conversion surgery (CS), defined as surgical resection performed when initially unresectable stage IV gastric cancer becomes amenable to R0 resection after a response to chemotherapy, has gained increasing attention [13,14,15]. In contrast, the phase III REGATTA trial showed that the nonselective addition of primary tumor resection did not improve survival [16]; thus, the value of CS depends on the selection of patients who can achieve R0 resection after a chemotherapy response. Indeed, the multicenter CONVO-GC-1 cohort and several retrospective studies have shown that patients who achieve R0 resection have a favorable prognosis, and R0 is the most consistent prognostic determinant [17,18,19]. Furthermore, the median OS of R0-resected patients has improved from approximately 24–36 months in the cytotoxic-chemotherapy era to over 48 months in the ICI/targeted era, and a framework consisting of approximately 6 months of preoperative systemic therapy, followed by R0 resection and up to 1 year of postoperative maintenance therapy, has been proposed [12,18]. Incorporating oligometastasis (limited metastatic disease responsive to systemic therapy) as an indication for CS, as well as refining its definition and indications, is also being advanced through the Bertinoro consensus and the European clinical practice guidelines for oligometastatic esophagogastric cancer (OMEC-4) [20,21].

In the ICI/targeted era, the number of patients undergoing CS after a first-line response has increased. Cohorts including ICI plus chemotherapy have shown higher conversion rates, high R0 rates, and pathological complete responses [22,23,24,25], and a high CPS has been associated with treatment response and successful conversion [22,23]. Reviews have summarized that the success of conversion is determined less by the type of regimen itself than by the depth and durability of the response to systemic therapy [12]. However, most prior reports were two-group comparisons (ICI plus chemotherapy vs. chemotherapy alone) or single-arm studies; data describing the real-world performance of CS—frequency, treatment outcomes, and prognosis—across the diverse contemporary first-line strategies of conventional chemotherapy, HER2-targeted therapy, and ICI within a single institution are limited.

Therefore, we classified our institutional AGC cohort into three groups according to the first-line regimen (conventional chemotherapy, trastuzumab-based, and ICI-based); descriptively compared CS rates, pathological response, surgical outcomes, and prognosis among the groups; and explored prognostic factors for OS. We further evaluated the validity of an extended definition of CS that includes oligometastasis and examined whether the factors associated with the depth of pathological response differ from those associated with prognosis. In addition, to place our findings in the context of the literature, we included a focused review summarizing CS cohorts in the ICI/targeted era. This study was an exploratory (hypothesis-generating) analysis conducted within the constraints of a single-center, retrospective, small-sample design.

## 2. Materials and Methods

### 2.1. Study Design and Patients

This was a single-center retrospective cohort study. The study included 187 eligible patients who began first-line systemic pharmacotherapy for unresectable AGC at our institution between January 2011 and November 2025. Survival was followed up until the data cutoff date of 10 June 2026. The year 2011 was selected as the cutoff because trastuzumab for HER2-positive cases was first administered at our institution in 2011 (this cohort included the trastuzumab group); therefore, earlier cases were not included.

Eligible patients were those with histologically confirmed gastric adenocarcinoma who were diagnosed with unresectable stage IV disease with distant metastasis (M1) at initial presentation (synchronous; post-gastrectomy recurrent cases were not included; Japanese Classification of Gastric Carcinoma [26]) and for whom the chemotherapy start date and survival outcomes were traceable. Exclusion criteria were: (i) histological diagnoses other than gastric adenocarcinoma (e.g., neuroendocrine carcinoma, gastrointestinal stromal tumor, malignant lymphoma, squamous cell carcinoma); (ii) missing data essential for analysis, including the chemotherapy start date, survival outcome, or disease stage; (iii) patients who did not begin first-line systemic chemotherapy (e.g., receipt of best supportive care alone); and (iv) an active concurrent malignancy within the preceding 5 years. Patient data were anonymized and managed using study-specific identification numbers; no personally identifiable information was included in the analysis dataset.

**Figure 1:**
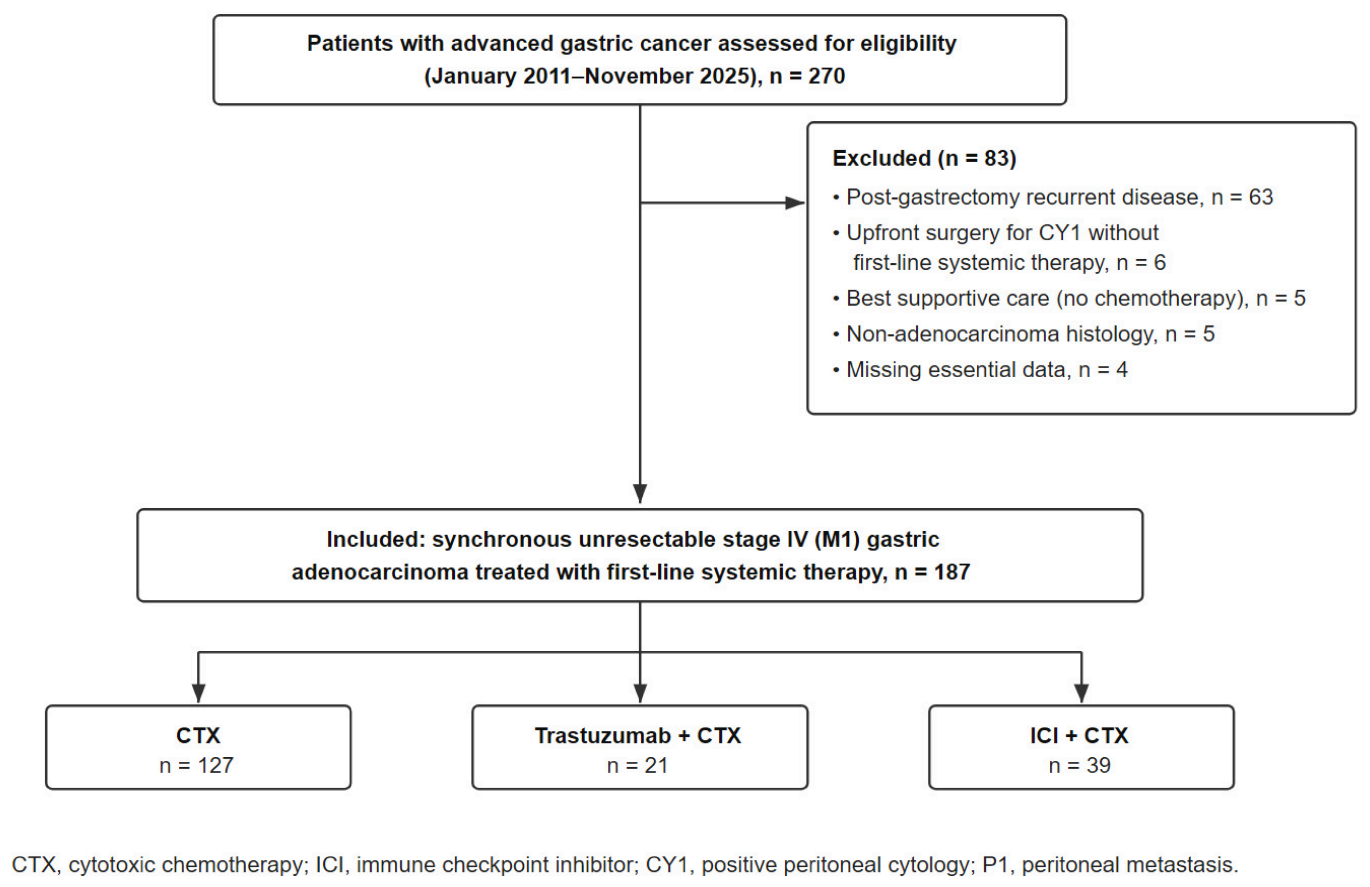
Patient flow diagram. Of 270 patients with advanced gastric cancer assessed for eligibility during 2011–2025, 187 with synchronous unresectable stage IV (M1) gastric adenocarcinoma who received first-line systemic therapy were included and grouped by first-line regimen. CTX, cytotoxic chemotherapy; ICI, immune checkpoint inhibitor; CY1, positive peritoneal cytology.

During the study period (January 2011–November 2025), 270 patients with advanced gastric cancer treated at our institution were assessed for eligibility. We excluded 63 patients with post-gastrectomy recurrent disease (metachronous), 6 who underwent upfront surgery for positive peritoneal cytology (CY1) without first-line systemic therapy, 5 who received the best supportive care without systemic chemotherapy, 5 with non-adenocarcinoma histology, and 4 with missing essential data (chemotherapy start date, stage, or survival outcome). The final analytic cohort comprised 187 patients with synchronous unresectable stage IV (M1) gastric adenocarcinoma who received first-line systemic therapy (Figure 1).

Positive peritoneal lavage cytology (CY1) was classified as M1 (stage IV) according to the Japanese Classification of Gastric Carcinoma; accordingly, patients with CY1 were included as unresectable stage IV. Four patients had positive peritoneal cytology (CY1) as the sole non-curative factor, without other distant metastasis, of whom two underwent conversion surgery; consistent with our extended definition, these were classified as oligometastatic conversion surgery. Staging laparoscopy was not performed routinely; it was undertaken selectively in patients with suspected serosal invasion on imaging, type 4 (scirrhous) tumors, or suspected peritoneal metastasis.

### 2.2. Grouping by First-Line Treatment

Patients were classified into three groups according to the first-line regimen: (i) conventional cytotoxic chemotherapy (CTX, *n* = 127), (ii) trastuzumab-based therapy (HER2-targeted; T-mab, *n* = 21), and (iii) ICI-based therapy (*n* = 39). Patients eligible for more than one category were assigned according to the following priority order: T-mab > ICI > CTX. Because HER2 positivity directly determines trastuzumab use, HER2-positive patients were classified into the T-mab group even when an ICI was co-administered. ICI use was defined as the presence or absence of nivolumab or pembrolizumab in the first-line therapy. The anti-Claudin18.2 antibody zolbetuximab is not an ICI and was therefore not included in the ICI group.

### 2.3. Definition of Conversion Surgery

CS was defined as surgical resection performed when an initially unresectable AGC became amenable to R0 resection after a response to first-line therapy [13]. In addition to conventional conversion, we included cases in which R0 resection was performed after a response to systemic therapy for oligometastatic disease (“Oligo” cases)—a solitary liver metastasis, para-aortic lymph node (No. 16a2/16b1) metastasis, or localized peritoneal metastasis (P1a; Japanese Classification of Gastric Carcinoma [26]), as well as positive peritoneal cytology (CY1) as the sole non-curative factor without other distant metastasis—within the definition of CS (total *n* = 74). These indications are consistent with the concept of oligometastasis proposed in the Bertinoro international consensus [20].

Gastrectomy with lymphadenectomy was performed according to the Japanese Gastric Cancer Treatment Guidelines, with D2 lymphadenectomy as the standard; the extent was individualized (D1+ in selected cases) according to tumor location, disease extent, and patient condition. Splenectomy with No. 10 nodal dissection was performed for advanced tumors of the greater curvature of the upper stomach. Combined resection of adjacent or metastatic organs (e.g., para-aortic nodal dissection, hepatic resection) was performed when necessary to achieve R0 resection.

### 2.4. Endpoints and Definitions

The primary endpoint was OS. OS was calculated from the date of chemotherapy initiation to death or the last confirmed survival date (calendar time). The secondary endpoints were CS rate, pathological response, and surgical outcomes (R0 resection rate and postoperative complications) in each group.

Pathological response: Assessed based on the histological treatment effect of the primary tumor (Ef grade, Japanese Classification of Gastric Carcinoma [26]) and ypStage. Grade 3 (corresponding to pathological complete response) and downstaging (ypStage 0/1) were compared between the groups.

Candidate prognostic factors: Baseline characteristics (age, sex, performance status [PS], body mass index); disease characteristics (number of non-curative factors, metastatic pattern, stage); tumor characteristics (histology, HER2 status, CPS, MMR status); treatment-related characteristics (first-line regimen, duration of preoperative chemotherapy, R0 resection); inflammatory–nutritional indices (neutrophil-to-lymphocyte ratio [NLR], prognostic nutritional index [PNI; 10 × serum albumin (g/dL) + 0.005 × total lymphocyte count (/µL)]; C-reactive protein-to-albumin ratio [CAR]); and tumor markers (CEA, CA19-9, CA125).

Cutoffs for continuous variables: Tumor markers were fixed at clinical standard values (CEA 5 ng/mL, CA19-9 37 U/mL, CA125 35 U/mL). The duration of preoperative chemotherapy was dichotomized at 6 months (180 days), following the cutoff used in CONVO-GC-1 [17]. For the other variables (age, NLR, PNI, and CAR), the optimal cutoff maximizing the log-rank statistic was used primarily, with robustness confirmed by sensitivity analyses using median and continuous values. Cutoffs derived by maximizing the log-rank statistic are susceptible to optimization (optimism/overfitting) bias; therefore, the corresponding subgroup hazard ratios were regarded as exploratory.

### 2.5. Statistical Analysis

Between-group comparisons used the χ^2^ test or Fisher’s exact test for categorical variables, and the Kruskal–Wallis test or Mann–Whitney U test for continuous variables. Survival was estimated using the Kaplan–Meier method, and between-group comparisons were performed using the log-rank test. Pairwise comparisons following a significant three-group test were adjusted for multiple comparisons using the Holm method. Follow-up duration was estimated using the reverse Kaplan–Meier method. Prognostic factors were assessed using univariable and multivariable Cox proportional hazards regression analyses.

The multivariable model was applied to CS cases, and clinically important covariates were entered based on the number of events (R0 resection, first-line ICI/trastuzumab therapy, and tumor burden/peritoneal disease status). Because the inflammatory–nutritional indices (NLR, PNI, and CAR) are inherently collinear, they were not entered simultaneously into the same model but were evaluated as “parallel models” in which each index was substituted individually into the same clinical covariates. The proportional hazards assumption was assessed using Schoenfeld residuals, and variables violating the assumption were addressed through stratification or a Kaplan–Meier approach.

Because the comparison between CS and non-CS cases is susceptible to immortal time bias, a landmark analysis was performed as a sensitivity analysis. The landmark time point was set a priori at six months to correspond to the typical duration of preoperative systemic therapy before CS in our cohort. Data preprocessing, descriptive statistics, exploratory Kaplan–Meier curves, and cutoff exploration were performed using Python (version 3.12; pandas 2.2.3, NumPy 2.2.2). Definitive survival and multivariable Cox analyses were performed using EZR (based on R version 4.6.0) [27]. A two-sided *p* < 0.05 was considered statistically significant.

### 2.6. Focused Review of the Literature

To place our institutional data in the context of the literature, reports of gastric cancer CS in the ICI/targeted era (including HER2-positive cases) were identified through a PubMed search. This review was designed as a focused narrative review rather than a comprehensive systematic review. Because the reports differed in their populations, definitions of CS, and treatment regimens, the data were not pooled; instead, treatment outcomes (conversion rates, R0 rates, pathological response rates, and prognosis) were compared descriptively alongside our institutional data (no meta-analysis was performed). Eligible reports were English-language cohort studies of gastric cancer CS during the ICI/targeted era (approximately 2019–2026) from which treatment outcomes could be extracted.

### 2.7. Ethics

This study was conducted in accordance with the Declaration of Helsinki and approved by the Institutional Review Board of Kanazawa University (approval number: 815063; approval date: 21 October 2025). Due to the retrospective study design, informed consent was obtained using an opt-out method through public disclosure of study information; therefore, individual written consent was not required. Trial registration was not required for this retrospective observational cohort study. Individual patient data are available from the corresponding author upon reasonable request, subject to privacy and ethical restrictions.

## 3. Results

### 3.1. Patient Characteristics

The 187 eligible patients were classified into three groups according to first-line treatment: CTX (*n* = 127), T-mab (*n* = 21), and ICI (*n* = 39) (Table 1). The three groups were comparable in terms of age, sex, PS, peritoneal metastasis, non-curative factors, and inflammatory–nutritional indices (PNI, NLR, and CAR). In contrast, the T-mab group was, by definition, enriched for HER2-positive disease (100%), had fewer undifferentiated tumors (21% vs. 66% in the CTX group and 65% in the ICI group, *p* < 0.001), and had more hematogenous (mostly hepatic) metastases (62% vs. 25–28%, *p* = 0.006), consistent with the biological features of HER2-positive gastric cancer. All patients in the ICI group started treatment in 2022 or later, reflecting a more recent cohort with shorter follow-up (death proportion 54%). CPS and MMR status were measured only in the ICI group.

### 3.2. Overall Survival by Group

**Figure 2:**
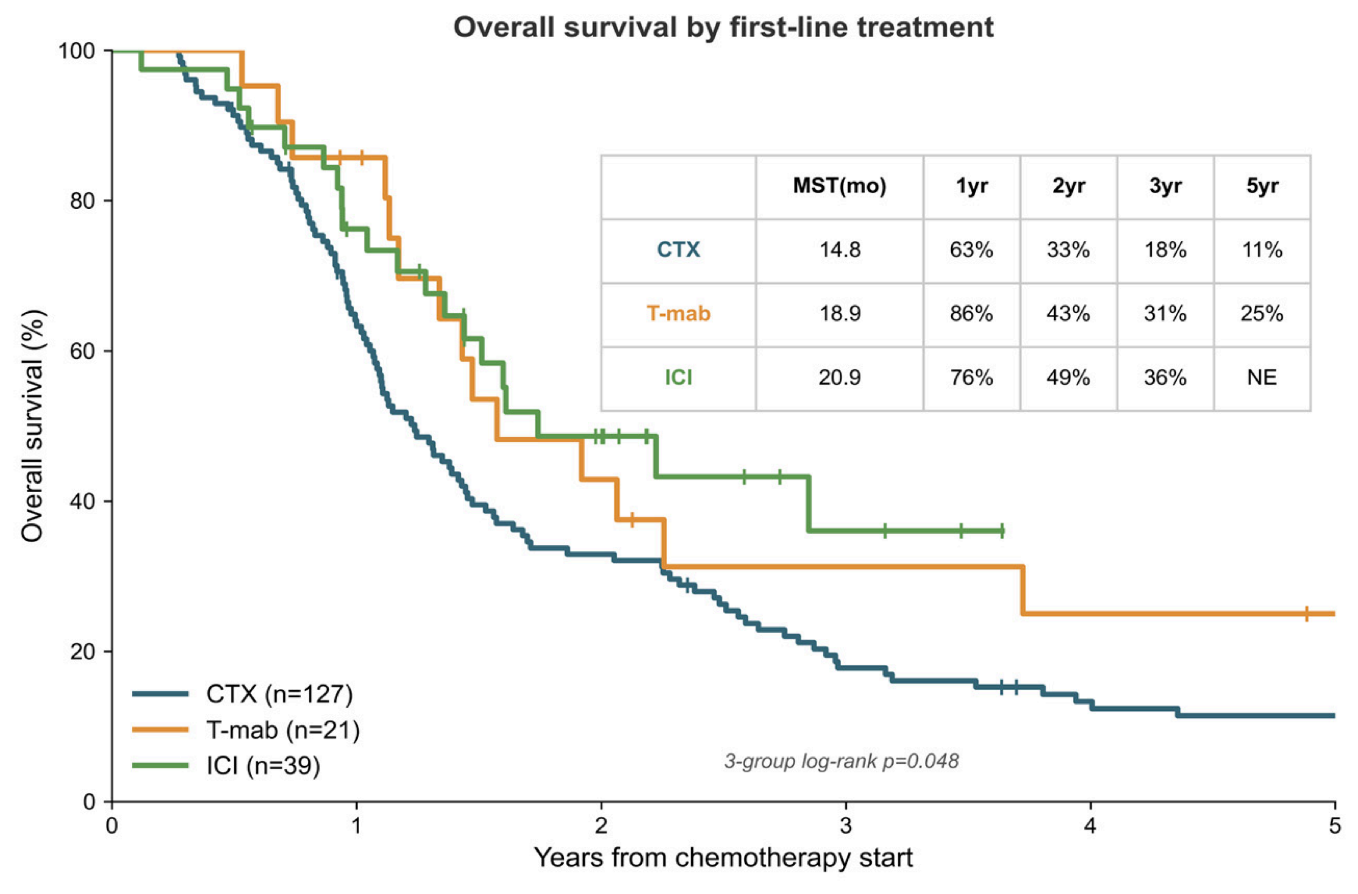
Overall survival by first-line treatment group (CTX/T-mab/ICI), Kaplan–Meier (*N* = 187). CTX, cytotoxic chemotherapy; T-mab, trastuzumab; ICI, immune checkpoint inhibitor, NE, not estimate.

The median follow-up period for the entire cohort was 61.7 months, as estimated by the reverse Kaplan–Meier method. Median OS was 14.8, 18.9, and 20.9 months in the CTX, T-mab, and ICI groups, respectively, with a borderline significant difference among the three groups (log-rank χ^2^ = 6.06, df = 2, *p* = 0.048; Figure 2). One-year OS rates were 63%, 86%, and 76%, and three-year OS rates were 18%, 31%, and 36%, respectively.

In the pairwise comparison, the ICI group had better OS than the CTX group before adjustment (unadjusted *p* = 0.041); however, this difference did not remain significant after Holm correction for multiple comparisons (adjusted *p* = 0.12). Comparisons between the CTX and T-mab groups (unadjusted *p* = 0.12) and the T-mab and ICI groups (*p* = 0.89) were also not significant.

Notably, patients in the ICI group started treatment in 2022 or later and therefore had a short observation period (median follow-up 26.3 months vs. 65.2 and 61.7 months in the CTX and T-mab groups, respectively; 54% deaths). Accordingly, the median survival time (MST) for the ICI group is likely immature, and the between-group OS difference should therefore be interpreted with caution given the confounding between the treatment era and follow-up duration.

To address era-related confounding, we compared OS within each group by period (before vs. from 2021). In the CTX group, median OS was essentially unchanged (14.9 vs. 14.4 months; log-rank *p* = 0.10), and the trastuzumab group showed no significant change (*p* = 0.5). Among patients treated from 2021 onward (*n* = 58), the three-group difference persisted (median OS: CTX 14.4, trastuzumab 17.2, and ICI 20.9 months; log-rank *p* = 0.01). Thus, a general secular improvement in outcomes did not explain the more favorable OS in the ICI group, although this should be interpreted cautiously given the small, selected recent-era subgroups with immature follow-up (see also the whole-cohort multivariable analysis, Section 3.4, in which neither era nor treatment group was independently significant).

### 3.3. Peritoneal Metastasis Subgroup Analysis (Whole Cohort)

**Figure 3:**
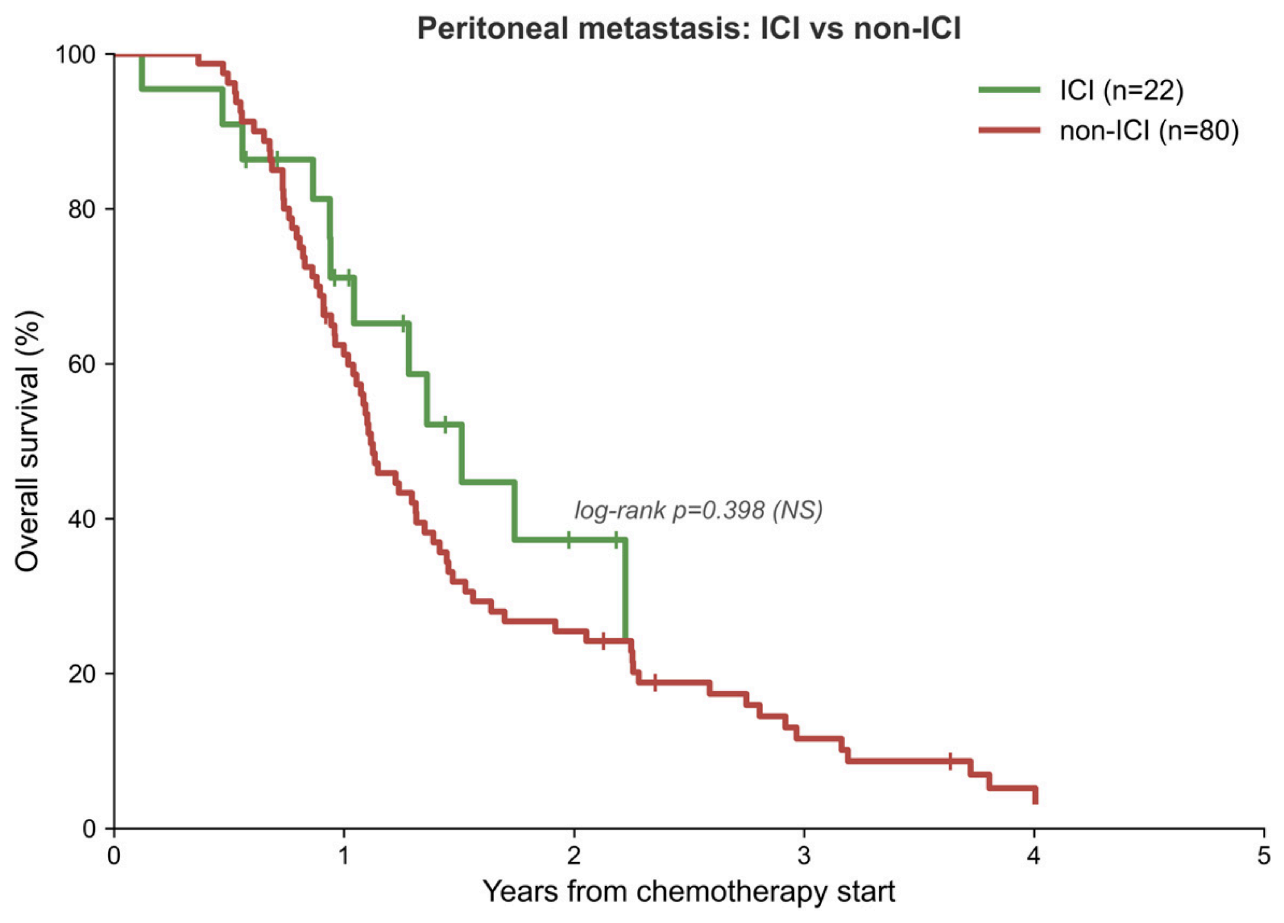
Overall survival in the peritoneal metastasis-positive subgroup by ICI use (Kaplan–Meier). ICI, immune checkpoint inhibitor.

Among patients with peritoneal metastasis (*n* = 102), there was no significant difference in OS between the ICI (*n* = 22, MST 18.1 months) and non-ICI groups (*n* = 80, MST 13.4 months) (*p* = 0.398; Figure 3). Although numerically slightly better in the ICI group, the difference was not significant, possibly owing to the limited sample size. In addition, peritoneal metastasis was associated with poorer prognosis in the univariable Cox analysis of CS cases (*n* = 74; hazard ratio [HR] 2.04; Section 3.8). Thus, patients with peritoneal metastasis continued to have unfavorable outcomes even in the recent ICI-era cohort, with no clear OS improvement from ICI use.

### 3.4. Prognostic Factors for Overall Survival in the Whole Cohort (Multivariable Cox)

Before focusing on conversion surgery (CS) cases, we performed a multivariable Cox analysis of OS in the whole cohort (*n* = 174 with complete covariate data; 135 deaths; concordance 0.72; Supplementary Table S1). CS was the strongest independent favorable prognostic factor (HR 0.26, 95% CI 0.17–0.40, *p* < 0.001). Multiple non-curative factors (HR 1.49, 95% CI 1.05–2.13, *p* = 0.026) and undifferentiated histology (HR 1.55, 95% CI 1.03–2.33, *p* = 0.034) were independent poor prognostic factors. The first-line treatment group (ICI or trastuzumab vs. CTX) was not independently significant after adjustment (ICI HR 0.65, *p* = 0.18; trastuzumab HR 0.62, *p* = 0.15), and era (year of chemotherapy initiation) was likewise not significant (HR 0.98 per year, *p* = 0.52). Thus, in the whole cohort, the survival difference was driven primarily by reaching CS rather than by the first-line regimen itself, which frames the CS-focused analyses that follow. However, because the CS group is inherently enriched for chemotherapy responders, the association between CS and survival is subject to substantial selection bias (by indication and treatment response); this whole-cohort analysis is therefore exploratory rather than causal, and the full model is presented as Supplementary Table S1.

### 3.5. Rate of Conversion Surgery

The CS rates were 41%, 33%, and 38% in the CTX, T-mab, and ICI groups, respectively, with no significant differences among the three groups (*p* = 0.794; Table 1). Thus, the rate of undergoing CS was comparable regardless of the first-line regimen, and the introduction of immunotherapy or targeted therapy was not associated with a higher CS rate.

### 3.6. CS Versus Non-CS Cases

**Figure 4:**
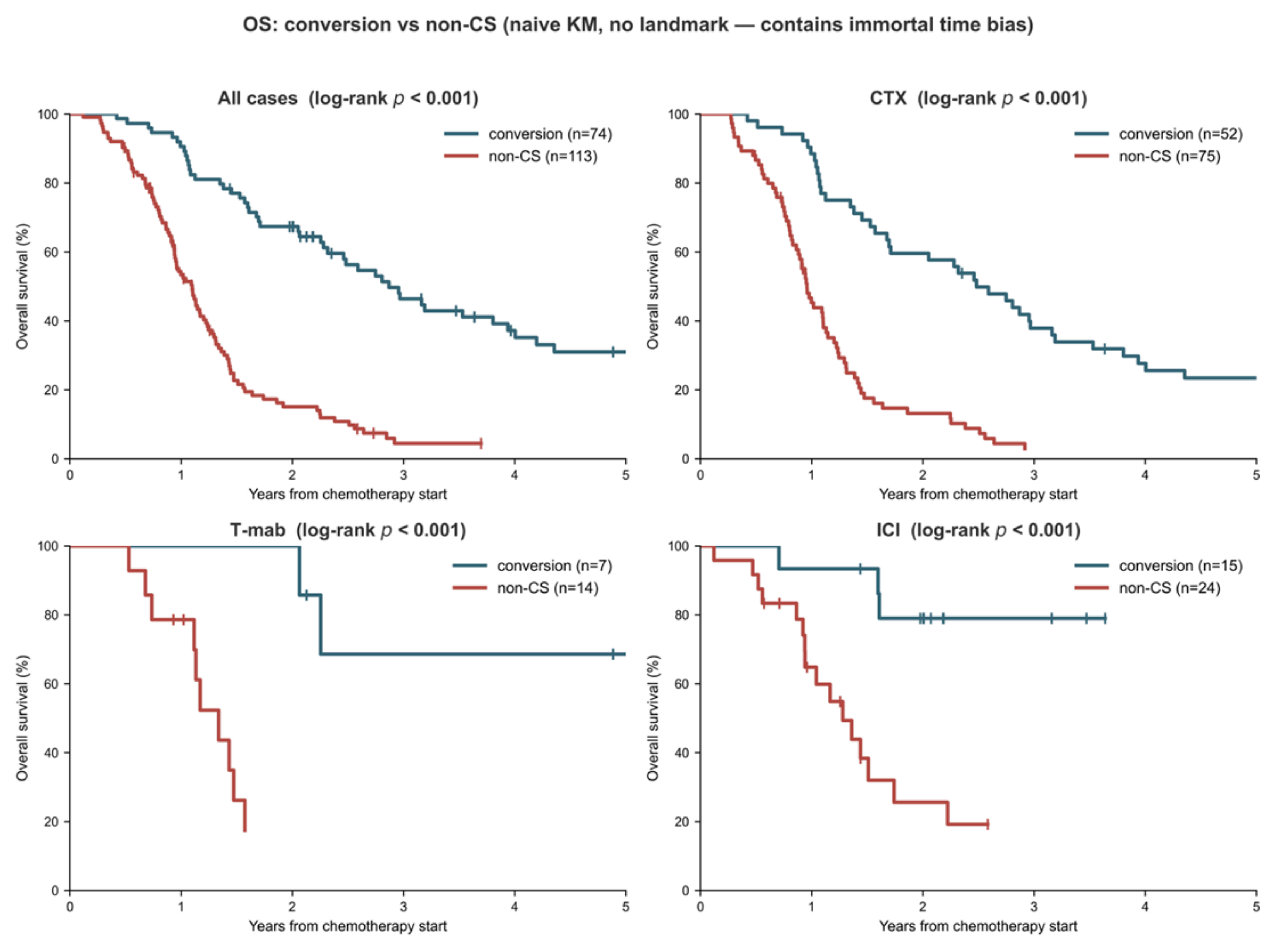
Overall survival of conversion surgery vs. non-conversion cases (Kaplan–Meier; subject to immortal time bias).

**Figure 5:**
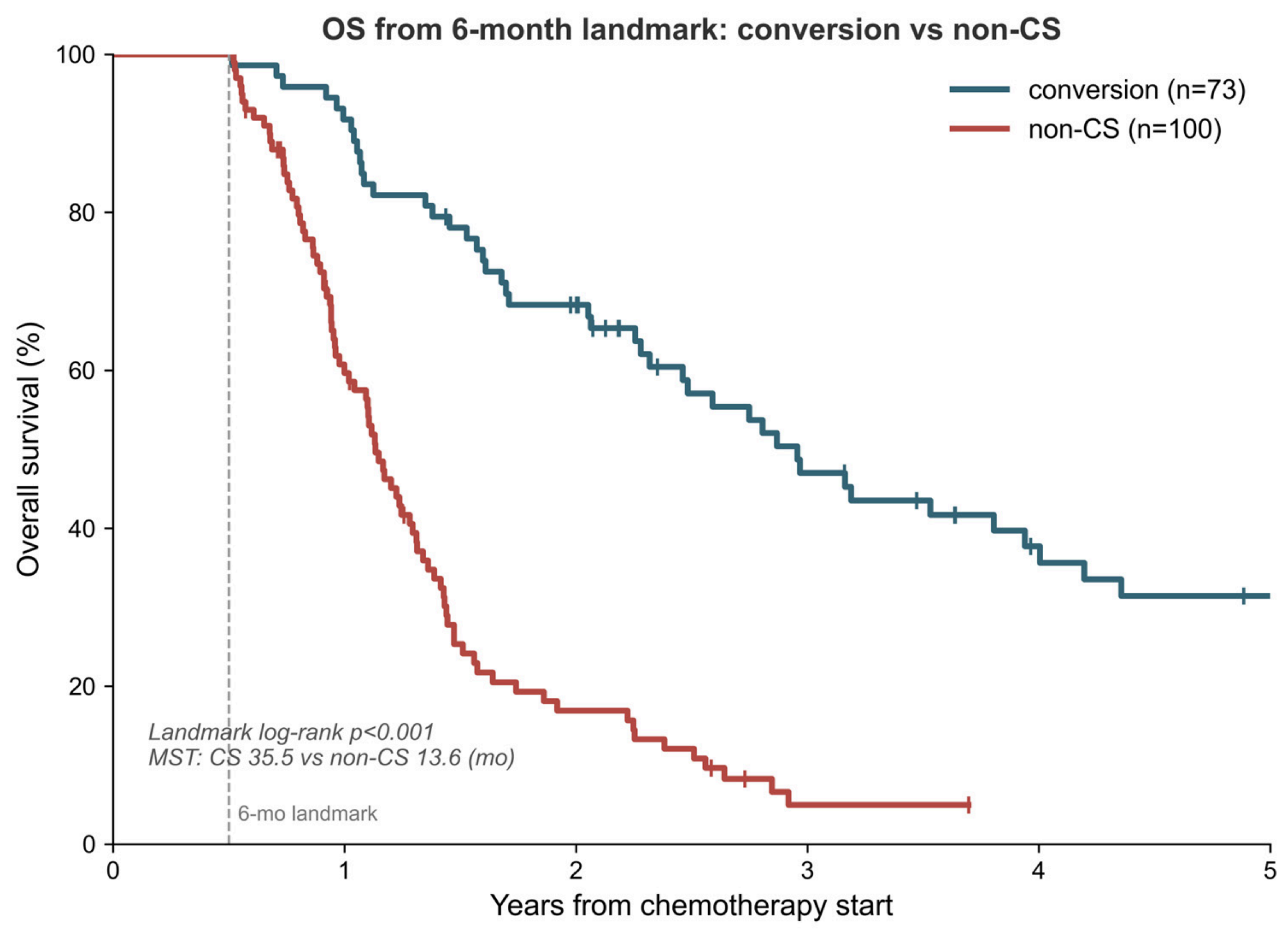
Landmark (6-month) overall survival of conversion surgery versus non-conversion cases (Kaplan–Meier).

In the unadjusted Kaplan–Meier comparison, patients who underwent CS had significantly better OS than those who did not (MST 34.4 vs. 13.2 months, *p* < 0.001; Figure 4). However, this comparison was susceptible to immortal time bias. In a landmark analysis at six months from chemotherapy initiation (patients alive beyond six months: CS, *n* = 73, non-CS, *n* = 100), OS remained significantly better among CS cases (MST 35.5 vs. 13.6 months; 2-year OS 68% vs. 17%; log-rank *p* < 0.001; Figure 5). Therefore, the favorable prognosis associated with CS was maintained even after correcting for immortal time bias, although residual indication selection bias precludes a causal interpretation.

### 3.7. Pathological Response, Surgical Outcomes, and Prognosis of CS Cases

Among the 74 patients who underwent CS (CTX, *n* = 52; T-mab, *n* = 7; ICI, *n* = 15; Table 2), pathological response was more pronounced in the T-mab and ICI groups. Histological grade 3 response rates were 2%, 29%, and 33% in the CTX, T-mab, and ICI groups, respectively, whereas downstaging rates to ypStage 0/1 were 10%, 43%, and 53%, respectively; both comparisons were statistically significant (both *p* < 0.001). In contrast, R0 resection rates were high in all groups (83%, 71%, 100%, respectively), with no between-group differences (*p* = 0.152), indicating that the curative resection rate among patients who reached CS was favorable regardless of the regimen.

**Figure 6:**
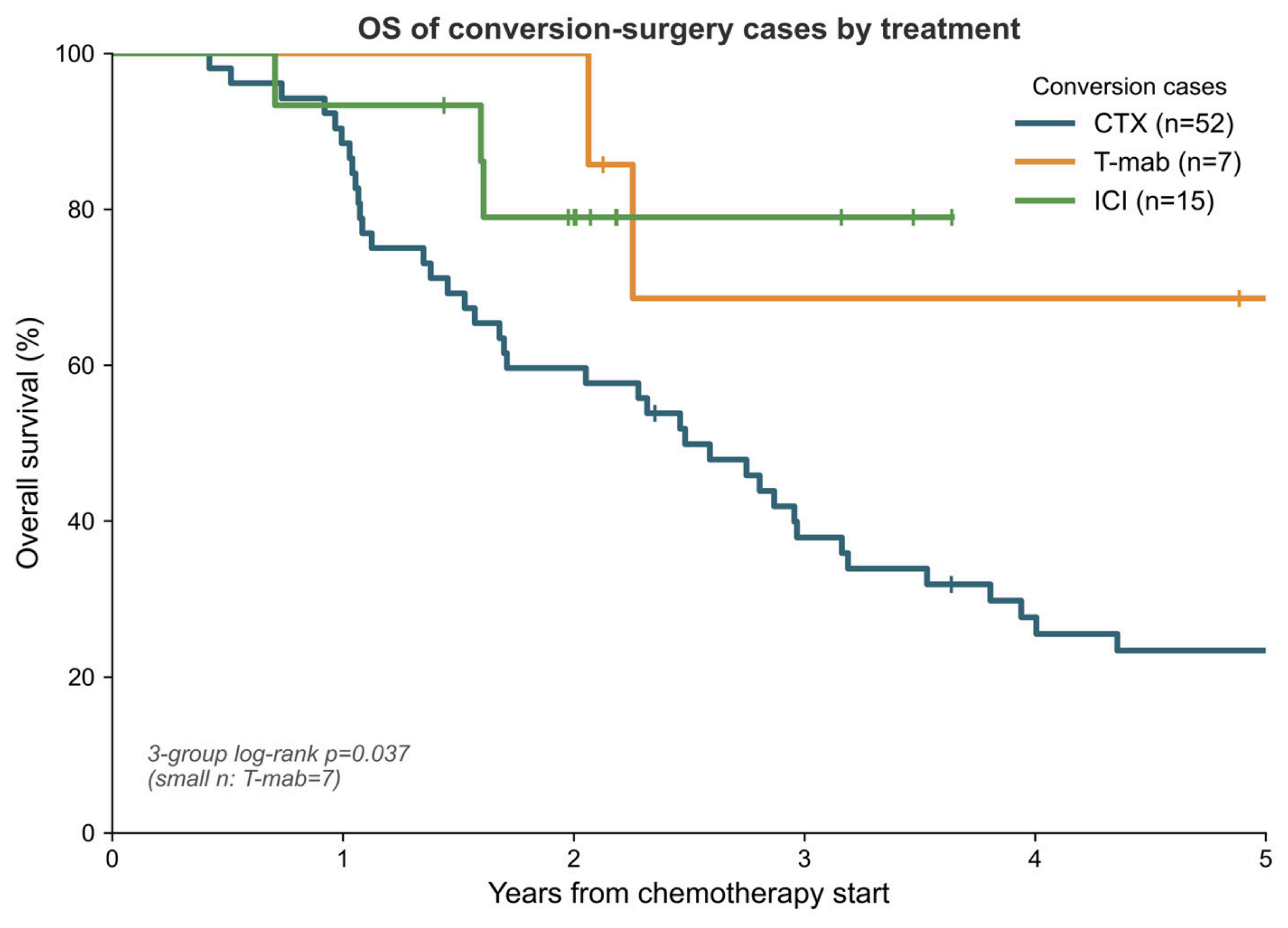
Overall survival of conversion surgery cases by first-line treatment group (Kaplan–Meier). CTX, cytotoxic chemotherapy; T-mab, trastuzumab; ICI, immune checkpoint inhibitor.

OS of CS cases differed among the groups (3-group log-rank *p* = 0.037). The two-year OS rates were 60%, 100%, and 79% in the CTX, T-mab, and ICI groups, respectively, and MST was 29.8, not reached, and 50.4 months, respectively (Figure 6). Notably, 13 of the 15 patients in the ICI group who underwent CS (87%) were nonperitoneal, indicating that ICI-driven conversion was concentrated in localized disease.

### 3.8. Prognostic Factors (Univariable and Multivariable Cox)

In the univariable analysis of CS cases (Table 3), R0 resection, ICI/T-mab therapy, and Ef grade 3 (a deep pathological response) were associated with a favorable prognosis, whereas ypT4, ypN positivity, multiple non-curative factors, peritoneal metastasis, signet ring cell histology, high NLR, and high CAR were associated with a poorer prognosis (undifferentiated type and low PNI showed trends). Because only one of the eight Ef grade 3 cases died (near-separation), Ef grade 3 was evaluated with a Firth penalized Cox model (HR 0.25, 95% CI 0.03–0.93, *p* = 0.036; log-rank *p* = 0.046). In contrast, the duration of preoperative chemotherapy (≥6 vs. <6 months) was not significantly associated with OS (HR 0.85, 95% CI 0.44–1.65, *p* = 0.635).

In the final multivariable model (with R0 resection, ICI/T-mab therapy, and peritoneal metastasis as covariates), R0 resection (HR 0.31, 95% CI 0.15–0.64, *p* = 0.002) and ICI/T-mab therapy (HR 0.37, 95% CI 0.15–0.90, *p* = 0.029) were independent favorable prognostic factors. Peritoneal metastasis was not significantly associated with prognosis (HR 1.47, *p* = 0.234). Because peritoneal metastasis violated the proportional-hazards assumption, a stratified model was also used, and the independence of R0 and ICI/T-mab was preserved.

The inflammatory–nutritional indices were evaluated in parallel models by adding each index individually to the same multivariable model. High NLR (HR 2.60, *p* = 0.005) and high CAR (HR 2.92, *p* = 0.002) were independent poor prognostic factors, whereas low PNI showed a similar directional association but was not significant (HR 1.92, *p* = 0.124).

The multivariable model was restricted to three clinically important variables (R0, ICI/T-mab, and peritoneal metastasis) to minimize overfitting, given the number of events (45 deaths; events per variable = 15 for the core three-variable model, and ≈11 for the parallel models, both ≥10). In sensitivity analyses adding ypT4, ypN, or Ef grade 3 individually, the independence of R0 and ICI/T-mab was preserved. Ef grade 3 could not be stably entered owing to near-separation.

### 3.9. Validity of the Extended CS Definition Including Oligometastasis

**Figure 7:**
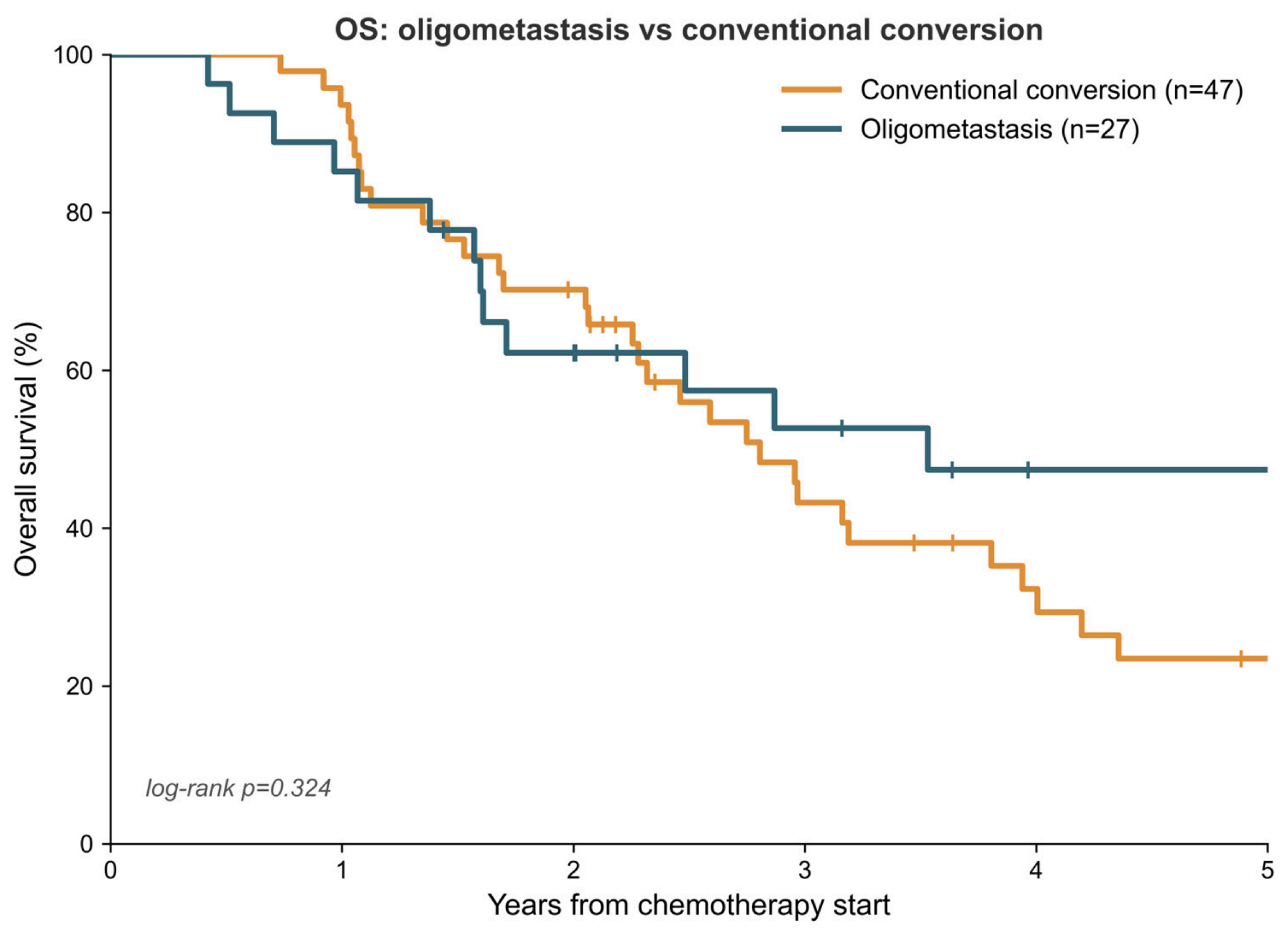
Overall survival of oligometastatic vs. conventional conversion surgery (Kaplan–Meier).

No significant difference in OS was observed between CS for oligometastasis (*n* = 27; median OS 42.4 months) and conventional conversion CS (*n* = 47; median OS 33.7 months) (*p* = 0.324; Figure 7). Among the Oligo cases, peritoneal lesions were limited (P1a) and the R0 rate was high. These findings support the validity of the extended definition of CS adopted in this study (conversion + oligometastasis).

### 3.10. Characteristics of Patients with a Deep Pathological Response (Grade 3) (Exploratory Analysis)

Of the 74 patients who underwent CS, eight had a pathological grade 3 (deep) response (CTX, *n* = 1; T-mab, *n* = 2; ICI, *n* = 5). Most of these patients (88%) received ICI or T-mab-based therapy. All patients with measured CPS data had CPS ≥ 5 (4/4). Peritoneal metastasis was uncommon (12%), oligometastasis accounted for 5 of 8 cases, and 43% were undifferentiated. In contrast, the inflammatory–nutritional indices did not differ between grade 3 and non-grade 3 cases (all *p* > 0.4).

These findings are exploratory and based on a few cases, and CPS and MMR status were measured only in the ICI group. Nevertheless, they provide the basis for the “two-axis” discussion below.

## 4. Discussion

This single-center exploratory cohort study described CS for AGC in the precision-medicine era according to first-line treatment strategy (cytotoxic chemotherapy, HER2-targeted therapy, or ICI-based therapy). Whereas the rate of undergoing CS was nearly comparable among the three groups, pathological response (grade 3, ypStage 0/1) was deeper in the trastuzumab and ICI groups. In the multivariable analysis, R0 resection and trastuzumab/ICI-based therapy were independent favorable prognostic factors, whereas host inflammatory–nutritional indices (NLR, PNI, and CAR) were independent poor prognostic factors. Taken together, the central interpretation of this study is a two-axis distinction in CS, in which the factors determining the “depth of pathological response” and those determining “prognosis” are partly different; response depth mainly reflects tumor biology, whereas prognosis reflects host factors and curative (R0) resection. Although each individual association has been reported previously, the contribution of this study is to show, within a single cohort, that the factor sets regulating response depth and prognosis are dissociable; deep and non-deep responders differed in tumor biology, but not in host inflammatory–nutritional indices (all *p* > 0.4).

To clarify the positioning of this study, we present in Table 4 the cohorts of gastric cancer CS reported in the ICI-targeted era, alongside our institutional data, as a focused, descriptive review; data were not pooled because the definitions, denominators, and regimens were heterogeneous. In recent cohorts, the CS rate ranges from approximately 30% to 50%, and the R0 rate from 70% to 100%; our findings align with these ranges (CS 33–41%, R0 71–100%). In contrast, the deep pathological response (pCR/grade 3) was high in recent cohorts, including ICI and HER2-targeted therapy (approximately 10–25%), higher than the cytotoxic-chemotherapy-era anchor (grade 3 ~5% in CONVO-GC-1). These are consistent with our finding that grade 3 rate was higher with ICI or T-mab (29–33%) than with CTX (2%). The prognostic signals that recur throughout Table 4 are threefold: (i) the outstanding weight of R0 resection; (ii) the poor prognosis conferred by residual-burden and disease-distribution factors such as peritoneal metastasis and ypN; and (iii) the regulation of response and conversion by biomarkers such as PD-L1 CPS and HER2—all consistent with the two-axis framework of this study. However, these reports differed greatly in their survival denominators (all patients, CS cases, and R0 cases) and follow-up. Therefore, a simple comparison of absolute values requires caution. Our group-wise MST of 14.8–20.9 months is for all AGC cases measured from chemotherapy initiation and cannot be compared with CS-restricted or R0-restricted survival values. This explanation is also the basis for our limited analysis as a focused review rather than a meta-analysis.

The significance of R0 resection as a prognostic determinant is consistent with that of previous reports. In CONVO-GC-1, the prognosis of R0 cases was strongly favorable [17,18], and in the multicenter study by Takeno et al. (*n* = 210), the MST of R0 cases was 41.5 months (vs. 20.7 months for R1/2) [28]. Importantly, once R0 was achieved, there was no prognostic difference according to the initial metastatic site (peritoneum, liver, or lymph node), and the independent prognostic factors were the residual pathological tumor burden, namely ypN positivity and ypT4 [28]. Similarly, in the Japanese 773-patient sub-analysis of CONVO-GC-1 by Yasufuku et al., the median OS of R0 cases was 47.8 months, and ypT and ypN were stronger prognostic predictors than the histological response of the primary tumor (as in the MAGIC trial); the favorable prognosis associated with prolonged preoperative chemotherapy was also interpreted as reflecting selection through the dropout of chemotherapy-resistant cases [18]. In our CS cases, the duration of preoperative chemotherapy (≥6 vs. <6 months) was not significantly associated with OS (HR 0.85, 95% CI 0.44–1.65, *p* = 0.635), consistent with this interpretation that the favorable prognosis of patients receiving prolonged chemotherapy reflects selection through the dropout of chemotherapy-resistant cases rather than the duration of chemotherapy per se. Our finding that R0 was the strongest independent favorable prognostic factor (HR 0.31) is consistent with these findings. A similar pattern recurs in recent full-text cohorts (Table 4): Li et al. (OS HR 0.20 for R0; among cStage IV R0 cases, no OS difference by the presence of MPR) [29], Liu et al. (R1/R2 the only independent unfavorable factor for EFS) [30], Liang et al. (for HER2-positive CS; peritoneal metastasis HR 20.8, ypN3 HR 8.95) [31], and Huang et al. (peritoneal metastasis HR 2.46) [23] all show this consistently. Overall, what divides prognosis after reaching surgery is the residual tumor burden (ypN/ypT4, ypN3) and disease distribution (particularly peritoneal), not the depth of response itself.

On the other hand, “which tumors respond deeply” was explained by tumor-biological factors. In this study, cases with a deep pathological response (grade 3) were predominantly ICI- and trastuzumab-treated (88%), all with measured CPS ≥ 5, with few peritoneal metastases and many oligometastases. The association of a high PD-L1 CPS with response and conversion is consistent with multiple prior reports [22,23,29]: in Liang et al., CPS ≥ 5 was an independent favorable factor for conversion success (OR 0.22) [22]; Huang et al. also reported higher conversion rates with CPS ≥ 1 (60.4% vs. 32.7%) and higher pCR with CPS ≥ 5 (22.2% vs. 0%), as well as high responses in dMMR/EBV-positive cases [23]; and in the multi-omics analysis by Li et al., MPR was associated with non-signet ring histology, high CPS, and high tumor mutational burden [29]. However, tumor biology is double-edged: in the same study by Liang et al., the only independent poor prognostic factor among CS cases was signet ring cell histology (HR 6.29; 7 of 9 R0-resected signet ring cases recurred) [22]. Thus, while “responsiveness markers” such as CPS/HER2 determine response and conversion, “malignancy markers” such as signet ring cells and undifferentiated histology influence prognosis after reaching surgery.

Importantly, the depth of response is not a prerequisite for a prognostic benefit. In the HER2-positive CS series by Arigami et al., all 12 cases reaching conversion achieved R0 but the pathological response was only grade 1a/1b (no grade 3), yet the 5-year OS was favorable at 68.6% (vs. 5.6% for chemotherapy alone) [32]; in the HER2-positive CS series by Hayano et al., the prognosis of R0 cases was likewise favorable [33]. R0-resected cases have a good prognosis even without a deep response, and the simple equation “depth of response = prognosis” does not hold. This supports the framework of this study, which separates response depth (tumor biology) from prognosis (selection, host, and R0).

Regarding the other axis governing prognosis, host factors (high NLR and CAR) were independent poor prognostic factors in our parallel models (low PNI was directionally similar but not significant). The association of inflammatory–nutritional status with the prognosis of CS/AGC is consistent with prior reports. Nakazawa et al. reported that no conversion was achieved in any high-risk case using the Gustave Roussy Immune Score (GRIm-s; calculated from neutrophil count, LDH, and albumin)—a conversion rate of 0%—and suggested that the GRIm-s could be a predictor of CS [25]. Palaj et al. also showed the prognostic significance of CEA, NLR, and PLR [34]. The additional finding of this study is that the inflammatory–nutritional indices, although poor prognostic factors, were not associated with the depth of pathological response (grade 3 vs. non-grade 3) (all *p* > 0.4). In other words, host inflammatory–nutritional status governs “prognosis” but does not predict “how deeply the tumor responds.” Integrating these, we propose as a hypothesis that CS candidates should be selected along two axes: the “likelihood” of a deep response and reaching conversion is estimated from tumor biology (biomarkers such as CPS/HER2 and disease distribution such as localized disease/oligometastasis), whereas the “prognosis” expected after reaching surgery is assessed from host inflammatory–nutritional status and the achievable R0 (as well as residual pathological tumor burden).

Among these prognostic determinants, peritoneal metastasis appears to remain an important and unresolved challenge, even in the ICI era. In this study, peritoneal metastasis cases showed no detectable OS improvement with ICI (Section 3.3), and 87% (13/15) of cases reaching ICI-driven conversion were non-peritoneal. Although our study did not assess the peritoneal microenvironment, these findings are consistent with the immunosuppressive microenvironment specific to peritoneal metastasis described in the literature. In gastric cancer peritoneal metastasis, cancer-associated fibroblasts (CAFs) have been reported to act as central regulators of a fibrotic, immunologically “cold” microenvironment, with a peritoneum-specific THBS2^+^ myofibroblastic CAF–SPP1^+^ tumor-associated macrophage axis implicated in immune exclusion and ICI resistance [35,36]. If this mechanism holds, current treatment centered on systemic ICI is unlikely to render peritoneal disease “convertible,” and bringing peritoneal metastasis within the reach of conversion surgery may require novel therapeutic strategies targeting the immunosuppressive peritoneal tumor microenvironment, such as CAF/fibrosis-axis targeting and intraperitoneal local therapy [37].

The validity of the extended CS definition is also consistent with this framework. No OS difference was observed between CS for oligometastasis and conventional conversion. In oligo cases, the peritoneal lesions were limited (P1a) and the R0 rate was high. The Bertinoro and OMEC efforts position oligometastatic disease as a localized condition responsive to systemic therapy and incorporate it into the indication for CS [20], consistent with Takeno et al.’s finding that prognosis after achieving R0 is unchanged by the metastatic site [28]; with a biology that is localized and amenable to R0, a prognosis comparable to conventional conversion can be expected even under the extended definition. The minimally invasive surgery (MIS) rate among CS cases was high in the recent group (ICI 87%); although the feasibility of MIS itself was reported before the ICI era [38], our study adds value by extending this across first-line treatment strategies and into the ICI era. However, the higher MIS rate in the recent group may also reflect the secular adoption of MIS. Loss of HER2 expression (HER2-loss; 29–61% after treatment resistance) during trastuzumab therapy [39,40] is a caveat in that the tumor-biology axis can change dynamically over the course of treatment.

This study has substantial limitations. First, it was single-center and retrospective. In particular, because the trastuzumab (*n* = 21) and ICI groups (*n* = 39), as well as the CS cases within each group, were few, the confidence intervals of the multivariable analysis were wide, and the deep response (grade 3, *n* = 8) could not be stably entered into the multivariable model owing to near-separation and was limited to description. Therefore, the two-axis distinction is hypothesis-generating rather than confirmatory. Second, because CS is selectively performed in patients with a good chemotherapy response and good general condition, the OS comparison between CS and non-CS cases carries a strong indication selection bias in addition to an immortal time bias, which even a landmark analysis may not be able to correct (presented as exploratory). Third, there was era-related confounding; patients in the ICI group started in 2022 or later with a short observation period, leading to favorably biased OS estimates, and staging (use of staging laparoscopy) and supportive care also changed over the study period (2011–2025). Fourth, CPS and MMR status were measured only in the ICI group; therefore, the association of biomarkers with response/prognosis could not be examined across the three groups, and the pathology was a single-center assessment that did not capture HER2-loss. Fifth, the comparison with prior reports was a non-systematic focused review; the data were not pooled because of heterogeneous definitions and regimens, and the study was not free from selective-citation bias.

In light of these limitations, the conclusions of this study are not definitive but hypothesis-generating. The proposed two-axis hypothesis—response depth governed by tumor biology and prognosis governed by host factors and R0—should be tested in prospective patient-selection studies integrating biomarkers (CPS/HER2 and, in the future, ctDNA and MMR) and host indices (inflammatory–nutritional status). Ongoing multicenter prospective trials (the JCOG2301 conversion study [41] and RENAISSANCE/AIO-FLOT5 [42]) will contribute to this validation. A quantitative synthesis of the evidence for CS after ICI will be conducted separately as a PRISMA-compliant systematic review/meta-analysis, distinct from the present narrative review (existing related SR/MAs: [43,44,45]).

**Table 4:** Gastric cancer conversion surgery in the ICI/targeted era: prior cohorts × the present study (focused review).

Study (First Author, Year)	Country/Design	Cohort (*N*)	First-Line Regimen	CS/Conversion	R0	pCR	Survival (Key)
ICI-based (mainly HER2−)							
Liang 2023 [22]	China/retrosp. multicenter	stage IV 136	ICI + chemo ± anti-HER2	CS 42 (≈31%)	90.5%	16.7%	1-yr OS 96.6%; signet ring HR 6.29; CPS ≥ 5 → conv OR 0.22
Huang 2025 [23]	China/retrosp. multicenter	stage IV 105	ICI + chemo	45.7%	83.3%	10.4%	3-yr OS 52.1% vs. 4.0%
Nakazawa 2024 [25]	Japan/retrosp. multicenter	104	nivolumab + chemo	11.5%	100%	—	high-risk GRIm-s → 0% CS
Hojo 2025 [24]	Japan/single-center	cIVB HER2− 103	nivolumab + chemo vs. chemo	28.6% vs. 8.0%	100%	—	CS MST NR vs. chemo 11.8 mo (*p* = 0.035)
Li 2025 [29]	China/retrosp. + omics	unresectable 73	ICI-based	surgery 82.2%	R0 HR 0.20	24.7%	OS 41.3 mo; TMB-H/KMT2D → response
Liu 2025 [30]	China/prospective ph II	stage IV 47	sintilimab + chemo + apatinib	R0 conv 51.1%	24/47	14.3%	OS 25.7 mo; R1/R2 only EFS factor
Nishi 2026 [46]	Japan/single-center	36 (ICI + chemo 34)	SOX + nivolumab	conv 11	100%	incl. pCR	2-yr OS 90%
HER2+/trastuzumab-based							
Liang 2025 [31]	China/retrosp. multicenter	HER2+ stage IV 232	trastuzumab ± ICI + chemo	CS 50	70.0%	22.0%	OS 50.9 vs. 22.0 mo
Arigami 2022 [32]	Japan/single-center	HER2+ 33	trastuzumab + chemo	CS 12	100%	grade 1a/1b only	5-yr OS 68.6% vs. 5.6%
Hayano 2020 [33]	Japan/case series	HER2+ 11	trastuzumab (1L)	CS 6	4/6	—	3-yr OS 66.7% vs. 20%
Cytotoxic-era prognostic anchors							
CONVO-GC-1 (2022; JP 2026) [17,18]	JP/KR/CN/retrosp. multicenter	1206; 773	chemotherapy	—	74%	~5% ¶	R0 median OS 47.8–56.6 mo; ypT4/ypN+ poor
Takeno 2024 [28]	Japan/retrosp. multicenter	CS 210	chemotherapy	R0 70%	70%	—	MST 32 mo; ypN+/ypT4 poor
Shin 2023 [47]	Korea/single-center	CS 118	CAPOX/HER2/ICI mix	—	(R0)	17.8%	BMI, HER2/MSI, targeted agents prognostic
Present study	Japan/single-center	AGC 187	CTX 127/T-mab 21/ICI 39	41%/33%/38%	83%/71%/100%	2%/29%/33%	MST 14.8/18.9/20.9 mo

Studies differ in eligibility, conversion/resectability definitions, denominator, regimens, and follow-up; values are descriptive and not pooled (focused review, not a meta-analysis). NR, not reached; CS, conversion surgery; pCR, pathological complete response; R0, margin-negative resection; OS, overall survival; MST, median survival time; ICI, immune checkpoint inhibitor; CPS, combined positive score; TMB, tumor mutational burden; EFS, event-free survival; GRIm-s, Gustave Roussy Immune Score. ¶ CONVO-GC-1: pCR shown as histological grade 3 (complete response), 60/1206 (5.0%); R0 median OS is 56.6 months in the full cohort [17] and 47.8 months in the Japanese subanalysis [18]. Per-study reference citations appear in Section 4.

## 5. Conclusions

In this single-center exploratory study, we describe the outcomes of CS for AGC in the precision-medicine era by first-line treatment strategy. Although the rate of reaching CS was comparable among the three groups, the pathological response was deeper with trastuzumab or ICI, and prognosis was dependent on R0 resection, treatment content (ICI or trastuzumab), and host inflammatory–nutritional status. The two-axis distinction—response depth mainly reflects tumor biology, whereas prognosis reflects host factors and curative resection—suggests a framework for selecting CS candidates that integrates biomarkers and host factors. In particular, peritoneal metastasis may remain a poor prognostic condition even in the ICI era, and the development of novel therapies targeting the immunosuppressive peritoneal microenvironment is a future challenge. These findings are hypothesis-generating and require validation in multicenter prospective studies.

## Figures and Tables

**Table 1:** Patient characteristics by first-line treatment group (*N* = 187). Continuous variables are median (IQR) (Kruskal–Wallis test); categorical variables are *n* (%) (χ^2^ test; for items with an expected count < 5 (PS ≥ 1, HER2, death), Fisher’s exact test). *p* values in Table 1 are descriptive and were not adjusted for multiple comparisons (exploratory). Abbreviations: CTX, cytotoxic chemotherapy; T-mab, trastuzumab; ICI, immune checkpoint inhibitor; IQR, interquartile range; BMI, body mass index; CEA, carcinoembryonic antigen; PS, performance status; HER2, human epidermal growth factor receptor 2; PNI, prognostic nutritional index; NLR, neutrophil-to-lymphocyte ratio; CAR, C-reactive protein-to-albumin ratio.

Variable	Overall (*N* = 187)	CTX (127)	T-mab (21)	ICI (39)	*p*
Age, median (IQR)	67 (59–72)	67 (60–72)	64 (56–68)	69 (62–76)	0.082
BMI	21.9 (20.1–24.3)	22.0 (20.0–24.0)	24.3 (21.4–26.0)	21.3 (20.2–22.3)	0.215
CEA (ng/mL)	3.3 (1.7–7.9)	3.4 (1.6–8.2)	4.0 (2.0–16.0)	2.5 (1.6–5.3)	0.398
CA19-9 (U/mL)	20 (8–126)	19 (8–109)	138 (23–851)	17 (6–54)	0.021
CA125 (U/mL)	21 (12–46)	23 (13–51)	25 (14–46)	13 (10–28)	0.100
PNI	44.7 (40.5–49.1)	44.2 (40.1–49.2)	44.0 (42.2–49.2)	45.4 (42.4–48.8)	0.801
NLR	3.2 (2.2–4.9)	3.1 (2.1–4.9)	3.6 (2.8–4.9)	3.5 (2.4–5.0)	0.904
CAR	0.10 (0.03–0.44)	0.10 (0.05–0.43)	0.13 (0.02–0.24)	0.08 (0.03–0.51)	0.669
Male sex	115 (64%)	79 (66%)	12 (60%)	24 (62%)	0.813
PS ≥ 1	20 (13%)	14 (15%)	2 (11%)	4 (10%)	0.779
Undifferentiated type	106 (61%)	78 (66%)	4 (21%)	24 (65%)	<0.001
HER2-positive	22 (12%)	1 (1%)	21 (100%)	0 (0%)	<0.001
Peritoneal metastasis	102 (55%)	71 (56%)	10 (48%)	21 (54%)	0.775
Hematogenous metastasis	58 (32%)	36 (28%)	13 (62%)	9 (25%)	0.006
Lymphatic metastasis	73 (39%)	49 (39%)	9 (43%)	15 (41%)	0.923
Multiple non-curative factors	56 (30%)	37 (29%)	9 (43%)	10 (26%)	0.358
Conversion surgery	74 (40%)	52 (41%)	7 (33%)	15 (38%)	0.794
Death	143 (76%)	108 (85%)	14 (67%)	21 (54%)	<0.001

**Table 2:** Rate and outcomes of conversion surgery by first-line treatment. The denominator for the CS rate is all patients in each group; the denominator for R0/pathological response/2-year OS/minimally invasive surgery (MIS) is CS cases (evaluable cases for each measure; grade 3 denominator for CTX is 51). *p* values are from the χ^2^ test; for items with an expected count < 5 (R0, grade 3, ypStage, MIS), Fisher’s exact test. ‡ The *p* value for 2-year OS is the 3-group log-rank for CS cases (χ^2^ = 6.6, df = 2). Abbreviations: CS, conversion surgery; CTX, cytotoxic chemotherapy; T-mab, trastuzumab; ICI, immune checkpoint inhibitor; R0, margin-negative resection; OS, overall survival; MST, median survival time; NR, not reached; MIS, minimally invasive surgery; ypStage, post-treatment pathological stage.

Measure	CTX (All 127, CS 52)	T-mab (All 21, CS 7)	ICI (All 39, CS 15)	*p*
CS rate (denominator = all in group, 127/21/39)	41%	33%	38%	0.794
R0 resection rate (CS cases)	83%	71%	100%	0.152
Grade 3 histological response (evaluable cases 51/7/15; 1 CTX case not evaluable)	2%	29%	33%	<0.001
Downstaging to ypStage 0/1 (CS cases)	10%	43%	53%	<0.001
2-year OS (CS cases; MST, months)	60% (29.8)	100% (NR)	79% (50.4)	0.037 ‡
Minimally invasive surgery rate (CS cases)	0%	29%	87%	<0.001

Note: the denominator for grade 3 and ypStage is CS cases (conversion + Oligo, 74; total grade 3 = 8 = CTX 1/T-mab 2/ICI 5).

**Table 3:** Cox regression for OS in CS cases (*n* = 74). The multivariable model uses R0, ICI/T-mab, and peritoneal metastasis as covariates; the inflammatory–nutritional indices were evaluated in parallel models adding each index one at a time to that model. HR (95% CI, *p*). Abbreviations: OS, overall survival; CS, conversion surgery; HR, hazard ratio; CI, confidence interval; R0, margin-negative resection; ICI, immune checkpoint inhibitor; T-mab, trastuzumab; NLR, neutrophil-to-lymphocyte ratio; PNI, prognostic nutritional index; CAR, C-reactive protein-to-albumin ratio; ypT4/ypN, post-treatment pathological T4/node-positive category.

Factor	Univariable HR (95% CI, *p*)	Multivariable HR (95% CI, *p*)
R0 resection (vs. R1/2)	0.32 (0.16–0.66, 0.002)	0.31 (0.15–0.64, 0.002)
ICI or T-mab (vs. CTX)	0.34 (0.15–0.81, 0.015)	0.37 (0.15–0.90, 0.029)
Preoperative chemotherapy ≥ 6 months (vs. <6)	0.85 (0.44–1.65, 0.635) ‡	—
Peritoneal metastasis	2.04 (1.13–3.71, 0.019)	1.47 (0.78–2.77, 0.234) †
ypT4	2.77 (1.49–5.14, 0.001)	— (sensitivity analysis)
ypN-positive	2.08 (1.09–3.98, 0.026)	— (sensitivity analysis)
Undifferentiated type	1.76 (0.94–3.29, 0.075)	—
Multiple non-curative factors	2.61 (1.15–5.92, 0.022)	—
Signet ring cell	4.39 (1.30–14.83, 0.017)	—
Ef grade 3 (marked response)	0.25 (0.03–0.93, 0.036) §	— (not entered; near-separation)
High NLR (≥5.1)	2.46 (1.28–4.71, 0.007)	2.60 (1.33–5.07, 0.005)
Low PNI (<39.5)	2.23 (0.99–5.03, 0.054)	1.92 (0.84–4.40, 0.124)
High CAR (≥0.216)	2.60 (1.41–4.79, 0.002)	2.92 (1.51–5.66, 0.002)

† Because peritoneal metastasis did not satisfy the proportional-hazards assumption (Schoenfeld residual test *p* = 0.013), it was also confirmed in a stratified model, in which R0 (HR 0.30, 95% CI 0.14–0.62) and ICI/T-mab (HR 0.38, 0.16–0.96) remained independent favorable prognostic factors. ‡ The 6-month cutoff for the duration of preoperative chemotherapy follows CONVO-GC-1 [17]. The HR and 95% CI are the definitive values from univariable Cox regression in EZR (the proportional-hazards assumption was satisfied; cox.zph *p* = 0.15). It was not entered into the multivariable model. § For Ef grade 3, only 1 of 8 cases died (near-separation); the univariable estimate is therefore from a Firth penalized Cox model (profile-likelihood HR, 95% CI, and *p* value; log-rank *p* = 0.046), as the ordinary Cox Wald estimate was unstable (HR 0.17, 95% CI 0.02–1.23, *p* = 0.079). It was not entered into the multivariable model.

## Data Availability

The data presented in this study are available from the corresponding author upon reasonable request. The data are not publicly available, owing to privacy and ethical restrictions.

## Supplementary Materials

The following supporting information can be downloaded at: https://www.mdpi.com/article/10.3390/cancers18152483/s1. Table S1: Multivariable Cox regression for overall survival in the whole cohort (*n* = 174, 135 deaths).

## Abbreviations

The following abbreviations are used in this manuscript:

AGC	advanced gastric cancer
CS	conversion surgery
CTX	cytotoxic chemotherapy
T-mab	trastuzumab
ICI	immune checkpoint inhibitor
OS	overall survival
MST	median survival time
PS	performance status
CPS	combined positive score
MMR	mismatch repair
Ef	histological treatment effect grade (Japanese Classification of Gastric Carcinoma)
NLR	neutrophil-to-lymphocyte ratio
PNI	prognostic nutritional index
CAR	C-reactive protein-to-albumin ratio
PLR	platelet-to-lymphocyte ratio
CEA	carcinoembryonic antigen
HR	hazard ratio
CI	confidence interval
MIS	minimally invasive surgery
pCR	pathological complete response
MPR	major pathological response
TMB	tumor mutational burden
CAF	cancer-associated fibroblast
GIST	gastrointestinal stromal tumor
IQR	interquartile range
