# Supplementary material for: Conversion Surgery for Advanced Gastric Cancer According to First-Line Treatment Strategy: A Single-Center Experience with a Focused Review of the Literature
Source: Cancers (Basel). 2026 Aug 2;18(15):2483. doi: 10.3390/cancers18152483 (PMC13465276; doi:10.3390/cancers18152483)
Supplement: Supplementary file 1 [file cancers-18-02483-s001.zip › cancers-4431544-supplementary.pdf]

cancers MDPI

Supplementary Materials

Table S1. Multivariable Cox regression for overall survival in the whole cohort (n=174, 135 deaths).

Variable	HR (95% CI)	*p*
Conversion surgery (vs none)	0.26 (0.17–0.40)	<0.001
ICI group (vs CTX)	0.65 (0.34–1.22)	0.18
Trastuzumab group (vs CTX)	0.62 (0.33–1.19)	0.15
Age (per year)	1.00 (0.99–1.02)	0.58
PS ≥1 (vs 0)	0.58 (0.30–1.13)	0.11
Peritoneal metastasis	1.32 (0.90–1.94)	0.16
Multiple non-curative factors	1.49 (1.05–2.13)	0.026
Undifferentiated histology	1.55 (1.03–2.33)	0.034
Era (per year from 2011)	0.98 (0.93–1.04)	0.52

n=174 after excluding 13 patients with missing covariate data; 135 deaths; concordance 0.72; likelihood ratio $p=3\times10^{-13}$. Reference for treatment group = CTX. Peritoneal metastasis showed a borderline proportional-hazards violation (Schoenfeld p=0.010); a stratified model yielded consistent results. A binary era term (from 2021 vs before) was also nonsignificant (HR 0.94, 95% CI 0.51–1.75, p=0.85). Abbreviations: HR, hazard ratio; CI, confidence interval; CS, conversion surgery; ICI, immune checkpoint inhibitor; CTX, cytotoxic chemotherapy; PS, performance status.
